# Seismic evidence of pop-up tectonics beneath the Shillong Plateau area of Northeast India

**DOI:** 10.1038/s41598-022-18389-0

**Published:** 2022-08-19

**Authors:** A. P. Singh, O. P. Mishra, O. P. Singh

**Affiliations:** 1 National Center for Seismology(NCS), Ministry of Earth Sciences (MoES), Lodhi Road, New Delhi, 110003 India; 2 Geological Survey of India (GSI), 27, J. L. Nehru Road, Kolkata, West Bengal 700001 India

**Keywords:** Natural hazards, Solid Earth sciences

## Abstract

Our detailed 3-D seismic tomographic assimilation using high-quality phase arrival time data recorded by the local seismographic network demonstrated that heterogeneities in the crustal faults have contributed significantly to the pop-up tectonics beneath the Shillong Plateau, characterized by high-V and low-σ. The major seismogenic faults, namely, the north-dipping Dapsi thrust in association with Dauki fault in the south and south dipping Brahmaputra fault in the north, located either side of the Shillong Plateau that acted as the causative factors for the pop-up, which attributed to the lithostatic (high-V, low-σ) and sedimentary (low-V, high-σ) load, respectively. Seismicity is found confined to a depth ≤ 60 km. Uneven distribution of structural heterogeneities in the upper crust is responsible for earthquake genesis of varying strengths. It is intriguing to note that high-velocity anomalies and low-ϭ in the uppermost crust, interpreted as the Shillong Plateau that acted as a geometric asperity and the juxtaposition of high-V and low-V became the source zone of the 1897 Shillong earthquake (M_s_ 8.7) as a novel observation for the region. Structural heterogeneities are distinctly distributed between low-V, high-σ and high-V, low-σ in the lower crust plays a major role for future intense seismogenesis due to differential strain accumulation.

## Introduction

**Figure 1:**
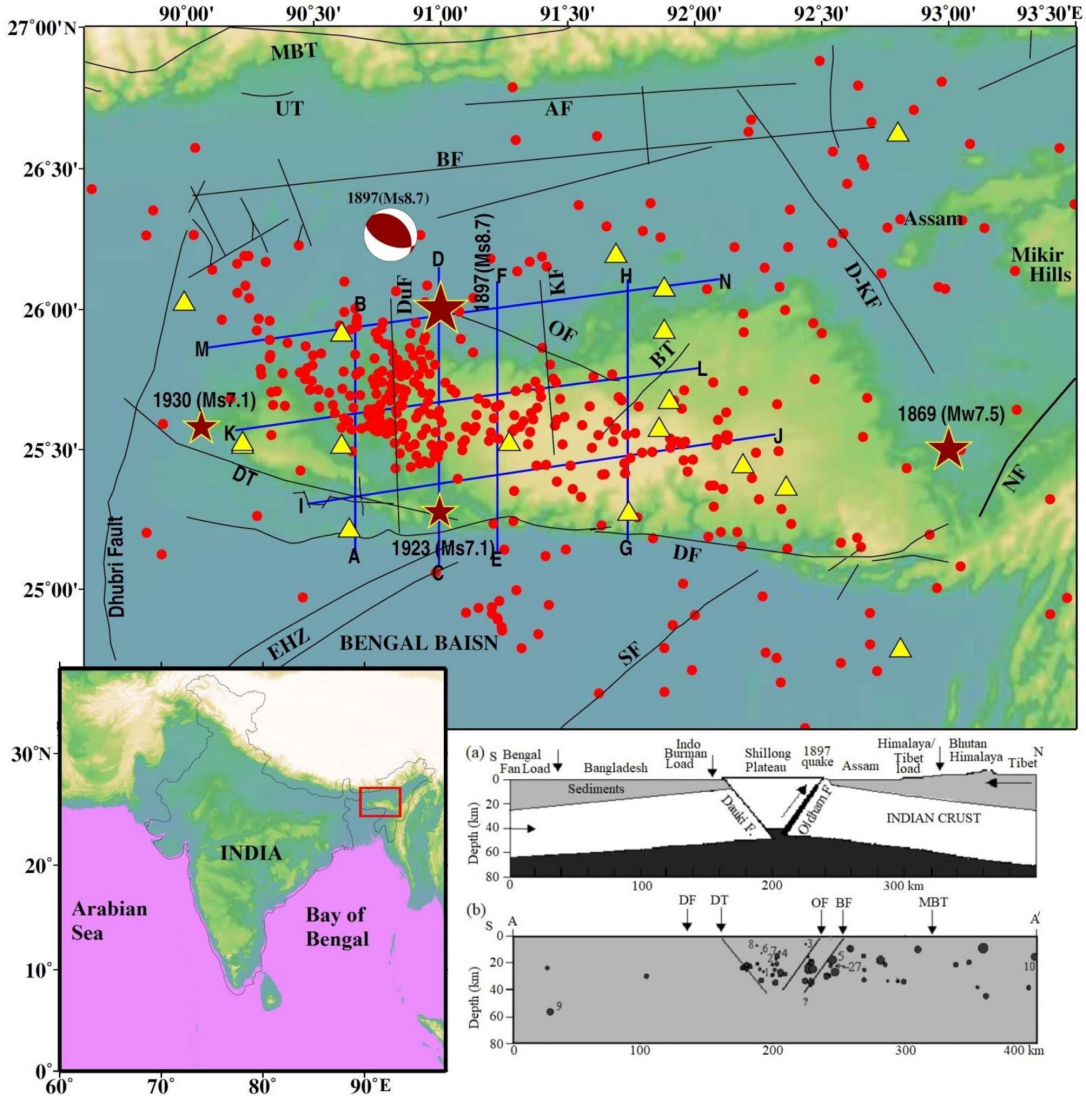
Topographic map of the Shillong Plateau showing major earthquakes (stars) M ≥ 7.0 including an 1897 great (M_s_ 8.7) earthquake. The focal mechanism of the 1897 earthquake is shown. The major tectonic features are also shown, such as DF: Dauki Fault; DT: Dapsi Thrust; OF: Oldham Fault; DuF: Dudhani Fault; KF: Kulsi Fault; BT: Barapani Thrust; BF: Brahmaputra Fault; NF: Naga Fault; D-KF: Dhansiri–Kopili Fault; SF: Sylhet Fault; UT: Ultapani Thrust; AF: Atherkhet Fault; EHZ: Eocene Hinge Zone and MBT: Main Boundary Thrust. The tectonic features are taken from the Geological Society of India, http://bhukosh.gsi.gov.in/Bhukosh/Public and from published literature. Triangles show the seismic station while red solid circles show the seismicity, used in the present study. Inset the location of the Shillong in the map of India (left panel) while right panel shows N–S section from Tibet to the Bay of Bengal showing the geometry of the plateau pop-up tectonic models proposed by (**a**) Bilham and England (2001) and (**b**) Kayal et al. (2012).

The plateau has attracted many geoscientists because of its unique location, complex geotectonic settings, frequent occurrence of earthquakes, a series of past damaging and non-damaging earthquakes and its continental type of structure. The Shillong Plateau experienced a great earthquake (M_s_ 8.7) on June 12, 1897, which caused catastrophic damage to life and property in the region^1–4^. The Shillong Plateau is a unique portion in the Precambrian history of the Indian shield that is detached from the peninsular shield during the Miocene, and moved horizontally by ~ 300 km eastwards along the E-W trending Dauki fault^5^. It lies at the boundary of the Himalayan arc to the north and the Burmese arc to the east. The Shillong Plateau is separated by the Dauki fault from thick Tertiary sediments of the Bengal basin in the south (Fig. 1). This plateau also over thrusts the Bengal Basin from the north^6^. The plateau, however, separates from the Himalayas in the north by the Brahmaputra River. The prominent E–W, N–S and NW–SE trending faults associated with the Shillong Plateau reflect complexity in the seismo-tectonics of the region (Fig. 1), which still remained inadequately investigated using geophysical and seismological tools because of several constraints, including inaccessibility of this region. The seismic activity in the Shillong plateau is categorised as a type of intra-plate seismicity, however, the seismicity pattern of the plateau region is not purely confined to the shallower layers like that of intra-plate shield, and rather earthquakes are also found locating ≥ 60 km depth^7–10^. The region also experienced two large devastating earthquakes, such as the 1923 Meghalaya (M_s_ 7.2) and the 1930 Dhubri (M_s_ 7.1)^9^.

The 1897 great earthquake was produced by a south-dipping hidden fault at the northern boundary of the Shillong plateau (Fig. 1); they named it Oldham fault that extends from a depth of ~ 9 km down to 35 km^4^. Bilham and England^4^ further suggested that the Shillong Plateau is a pop-up system confined by the two reverse faults, namely a south-dipping hidden Oldham fault in the north and a north dipping Dauki fault in the south^4,11^. Furthermore, based on the best fitting solution for minimizing the misfit of the angular changes and their uncertainties, Bilham and England (2001) advocate for a slip of 16 m on a fault plane striking ESE for 110 km and dipping correspondence to 57° with a rake of 76° beneath the northern edge of the plateau (Fig. 1). Furthermore, the Dauki fault is the surface exposed southern bounding fault. It is ~ 194 km long and dips 50^o^N with a strike of 270°.

Rao and Kumar^12^, however, suggested the pop-up tectonics of the Shillong Plateau, and they argued that the pop-up mechanism is facilitated by the Dauki fault to the south, Brahmaputra fault to the north, Dhubri fault to the west and Disang thrust to the east (Fig. 1). Rao and Kumar^12^ and Nandy^13^ defined the E–W segment of the Brahmaputra River to the north of plateau as the Brahmaputra fault. The Dauki fault is a near vertical gravity fault or a southerly dipping strike-slip/normal fault, not a north-dipping thrust fault as envisaged in the pop-up tectonic model^5,13^. The south dipping structure is conformable with the E–W segment of the Brahmaputra fault at the northern boundary of the plateau. Recently, Kayal et al.^14^ opined that the surface projection of the Oldham fault and the surface trace of the Brahmaputra fault are very close, within ~ 20 km from the epicentre of the 1897 great earthquake (M_s_ 8.7). The geological field investigation, however, argued that the Dapsi thrust seems to be north-dipping but geologically, geomorphologically and topographically there is no evidence of the Oldham fault or the Brahmaputra fault^14^. The epicentral area impacted during the 1897 Shillong earthquake and subsurface rupture is shown in supplementary material (Fig. S1), which was reported by Oldham’s (1899).

The fault bounding pop-up structure penetrates into the whole crust^4^. The large difference in basement depth between the Shillong Plateau and the Bengal basin cannot be at all explained by a thrust movement^13^. The micro-earthquake data, however, suggest that the Dapsi thrust, western extension of the Dauki fault, is north-dipping and seismogenic^15^. The 1897 great earthquake occurred on the detachment, and the rupture propagated to the south^16^. Earlier investigations in the study region were based on either limited data set for the limited period or regional data collected from the seismological catalogues ascribed to the International Seismological Centre (ISC)^17,18^. The ISC data has neither available small earthquake parameters nor accuracy for direct or converted phase arrivals.

Kayal and Zhao^19^ used limited data sets recorded in a limited seismic network to determine the 3-D seismic structure of the region. However, due to complexity in the perspective of seismotectonics and geodynamics, no one is able to provide a single tectonic model to explain the complex structure and occurrence of shallow and scattered earthquakes (Fig. 1) for this region^19–22^. It also appears that this plateau, although it generated a large earthquake in the past, is quite poorly understood due to the paucity of data arising from the inadequate deployment of seismological networks in this region. The earthquake patterns are quite different in the western and eastern part; however, the overall seismic activity of the region may be assumed to depend on the stress generated and resistance offered by compression from all sides in the regions. Despite several studies conducted in the study region^10,14,15,21,23,24^, no common consensus has yet been cropped out to address the key issue of the active seismogenesis of the plateau and its area. There is also still a common consensus over the origin and existence of the Oldham fault and pop theory proposed by Bilham and England^4^. The assimilated three-dimensional seismic structures of the Shillong Plateau derived from high-quality P- and S- wave arrival time data from precisely and closely located earthquakes recorded at dense seismographs yield reliable crustal models and allow us to answer several outstanding questions in the study region.

We, therefore, in this study, an attempt has been made to examine by delineating and detecting the plausible causative faults responsible for causing the pop-up tectonic in the Shillong Plateau region by conducting detailed 3-D local scale seismic tomography based on very high quality of the resolvable data with aim of imaging structural heterogeneities associated with causative faults. We also attempted to characterize the source zones of the past damaging earthquake.

## Results

**Figure 2:**
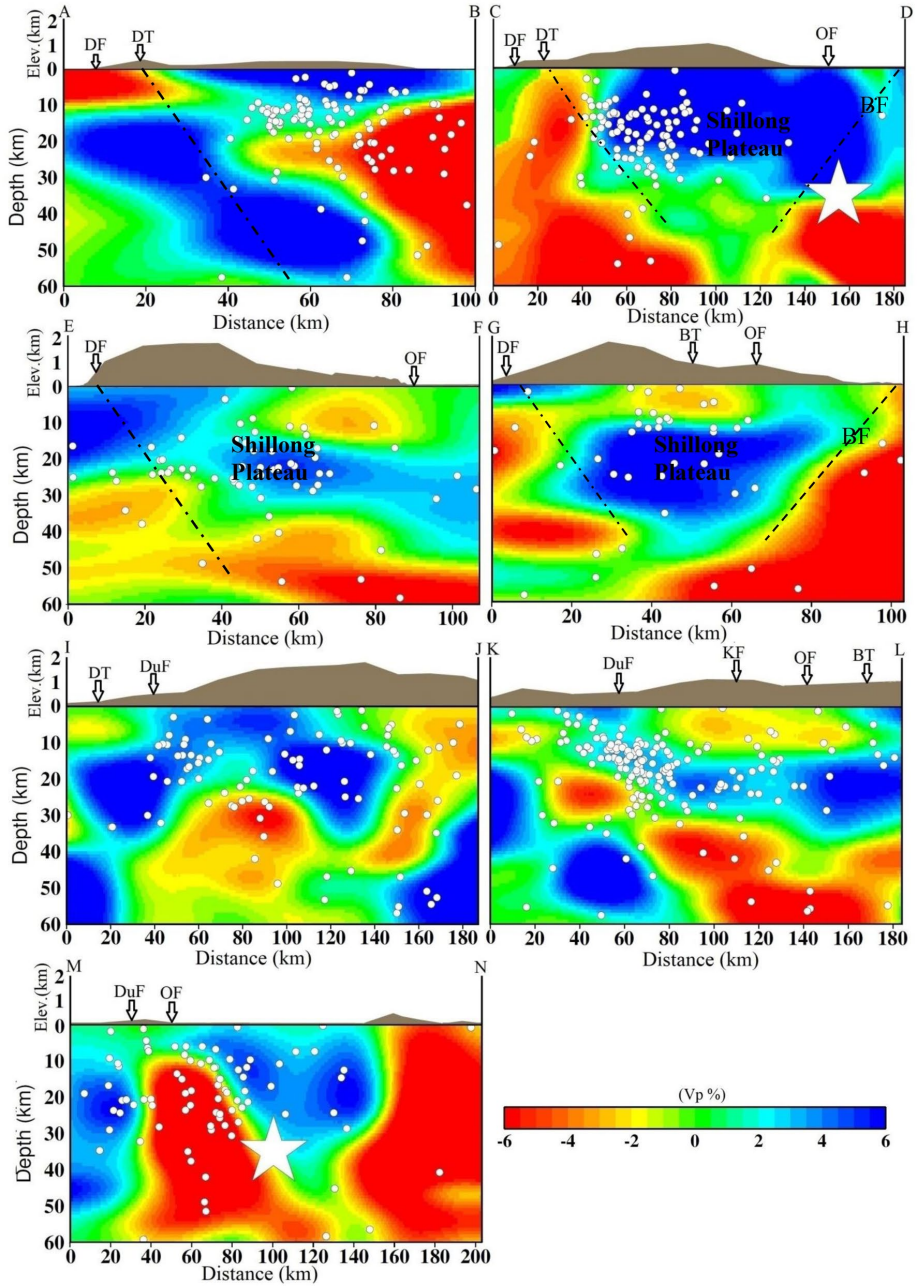
Vertical cross sections of the P-wave velocity models. The positions of the cross sections are shown in Fig. 1. The star shows the 1897 Shillong earthquake (M_s_ 8.7). The geological faults are also marked.

**Figure 3:**
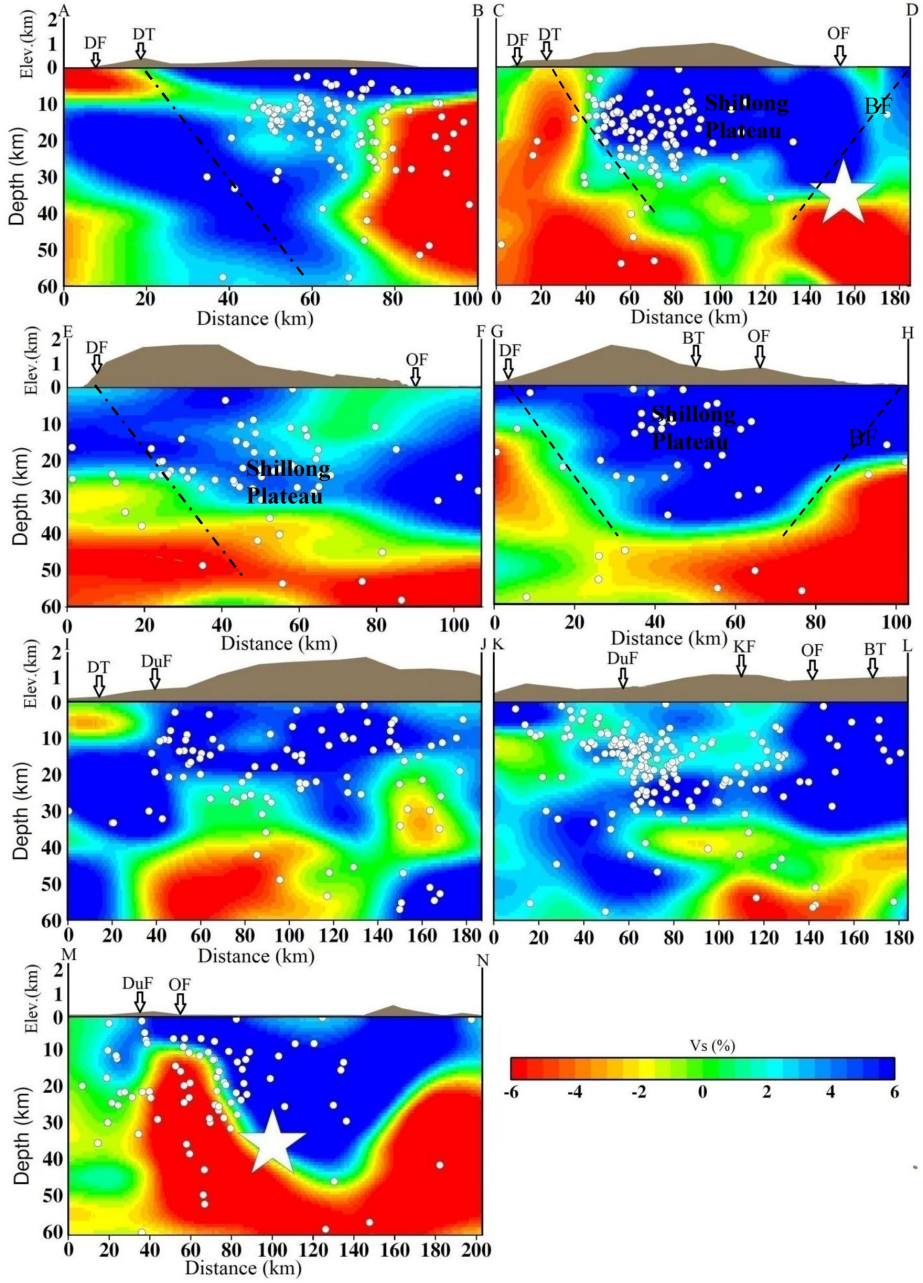
Vertical cross sections of the S- wave velocity models. The positions of cross sections are shown in Fig. 1. The star shows the 1897 Shillong earthquake (M_s_ 8.7). The geological faults are also marked.

**Figure 4:**
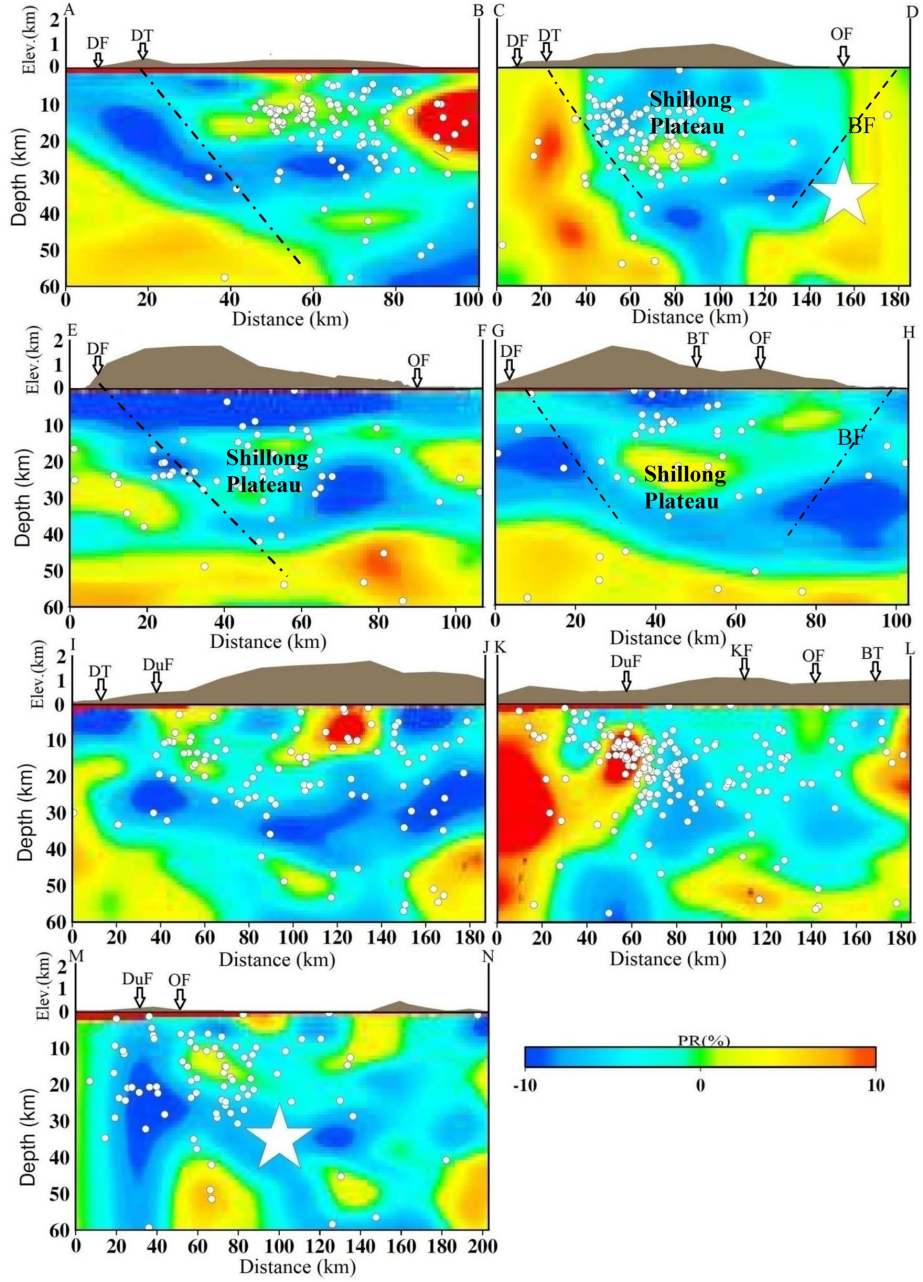
Vertical cross sections of the σ models. The positions of cross sections are shown in Fig. 1. The star shows the 1897 Shillong earthquake (M_s_ 8.7). The geological faults are also marked.

The results of the synthetic test suggest that the checkerboard pattern is well recovered alternatively for both V_p_ and V_s_ structures for all depth levels because of the concentration of high-quality data recorded at a dense seismic network that causes a high density of ray coverage at all depths as evident from hit count tests. However, the velocity images (V_p_, V_s_) and Poisson’s ratio (σ) also have comparatively lower resolution for velocity images assimilated between 50- and 60-km depth layers because of the lower number of events from these layers, reducing hit counts at these depths. Tomographic inversions and resolution tests with different grid spacing, initial velocity models, and datasets were also performed. We also conducted several changes in the velocity gradient and velocity values of the starting model. Although there were some small changes in the amplitude of the velocity anomalies (< 1.0%), the patterns of the tomographic images were generally the same. The overall patterns of the V_p_ and V_s_ images are similar, but there are some differences in details. The seismicity in the study area is mostly confined within the middle portion in two seismically active segments: N–S trending along the Dudhnoi fault and NE-SW trending along the Barapani Thrust (Fig. 1). The establishment of a denser seismographic network in comparison to the previous study^19^ provided significant variations in terms of seismic velocities (V_p_ and V_s_) and Poisson’s ratio (ϭ) at different depths in the study region that may be related to the subsurface crustal heterogeneities (Figs. 2, 3, 4, Supplementary materials, Figs. S2 and S3a,b) that can have appreciable control on the lithostatic stress pattern of the region^25^.

We examined vertical cross sections of the seismic velocities and Poisson’s ratio (σ) structures along seven selected profiles, four along the north–south directions (Profiles A–B, C–D, E–F and G–H) and three along the west–east directions (Profiles I–J, K–L and M–N) of the Shillong Plateau, which are shown in Fig. 1. We examined these cross sections to understand the lithospheric structures and geometry of faults at different depths (Figs. 2, 3, 4, Supplementary Fig. S2). Our results reveal strong lateral structural heterogeneities within the top 60 km in the study area. The broad V_p_- and V_s_- structures remain the same although some small patterns have changed in all the profiles. We also examined Vp/Vs ratio cross-sections along the selected profiles (Supplementary Fig. S2). In the profiles along the study area, we observed a prominent low σ zone down to a depth of 40 km. Furthermore, higher σ was observed at deeper depths > 40 km. The study areas are, however, well covered by the seismological network; the seismic data produced high-resolution seismic structures (Supplementary Fig. S4a,b). It is clearly observed that the Dauki fault, Oldham fault and Barapani thrust are located at the boundaries of low and high velocities. Mostly, seismicity is associated with high-V_p_ and high-V_s_ and low-σ. A robust feature appears in both V_p_- and V_s_- as well as σ- cross sections and we found that the Dauki fault gently dips northward toward high-V_p_ and -V_s_ and the low-σ layer beneath the Shillong Plateau down to a depth of 50 km (Figs. 2, 3, 4). This northward dip velocity and layers increase gradually and penetrate the uppermost mantle. Such high-velocity layers at deeper depths have also been revealed in Himalayan segments^26^. We observed that the earthquakes are mostly confined within a depth of 40 km. It is also noted that there is scattered or less seismic activity at deeper depths. These results indicate rheological changes in the crust at the Moho depth. The high velocity structures are all along the north direction compared to the south direction down. High seismic velocities are identified between two low velocities along cross sections as high velocities compared to the Shillong plateau. High velocities area is observed on the western side with a number of smaller magnitude earthquakes in the entire investigation area than in the eastern portion of the surveyed area^14^.

The velocity structures and their bearing on the nature of the lower crust are clearly reflected (Figs. 2, 3, 4, see the profile locations in Fig. 1). The V_p_- and V_s_-structures show that high seismic velocity anomalies are bounded by low seismic velocity anomalies, consistent with a the Shillong Plateau bounded by the Dapsi fault and the Brahmaputra Fault. It may be inferred that the movement along these two faults might be coupled due to differential seismic velocity anomalies in the region, thus turning the local faults and lineaments active and generating seismic activity of smaller magnitudes at shallower depths. It may be stated that a differential stress conditions is observed in the entire investigation area, and the western part is less stressed with a number of smaller magnitude earthquakes than the eastern part of the surveyed area^14^. Along the profiles, the high velocities and low-ϭ and zones in the Shillong reflect the plateau and are well imaged at depths (Figs. 2, 3, 4). The low-V_p_ and -V_s_ zones and high-ϭ at shallower depths (2–10 km) may be attributed to fractures or sediment vertical structures, which are seismically active. In the E-W profiles, the lateral variations in seismic velocities and velocity ratios are more or less similar. Interestingly, the 1897 earthquake occurred at the junction of high and low velocities and low σ at the depth of ~ 35 km (Figs. 2, 3, 4).

### Pop-up tectonics beneath the Shillong Plateau and Discussion

**Figure 5:**
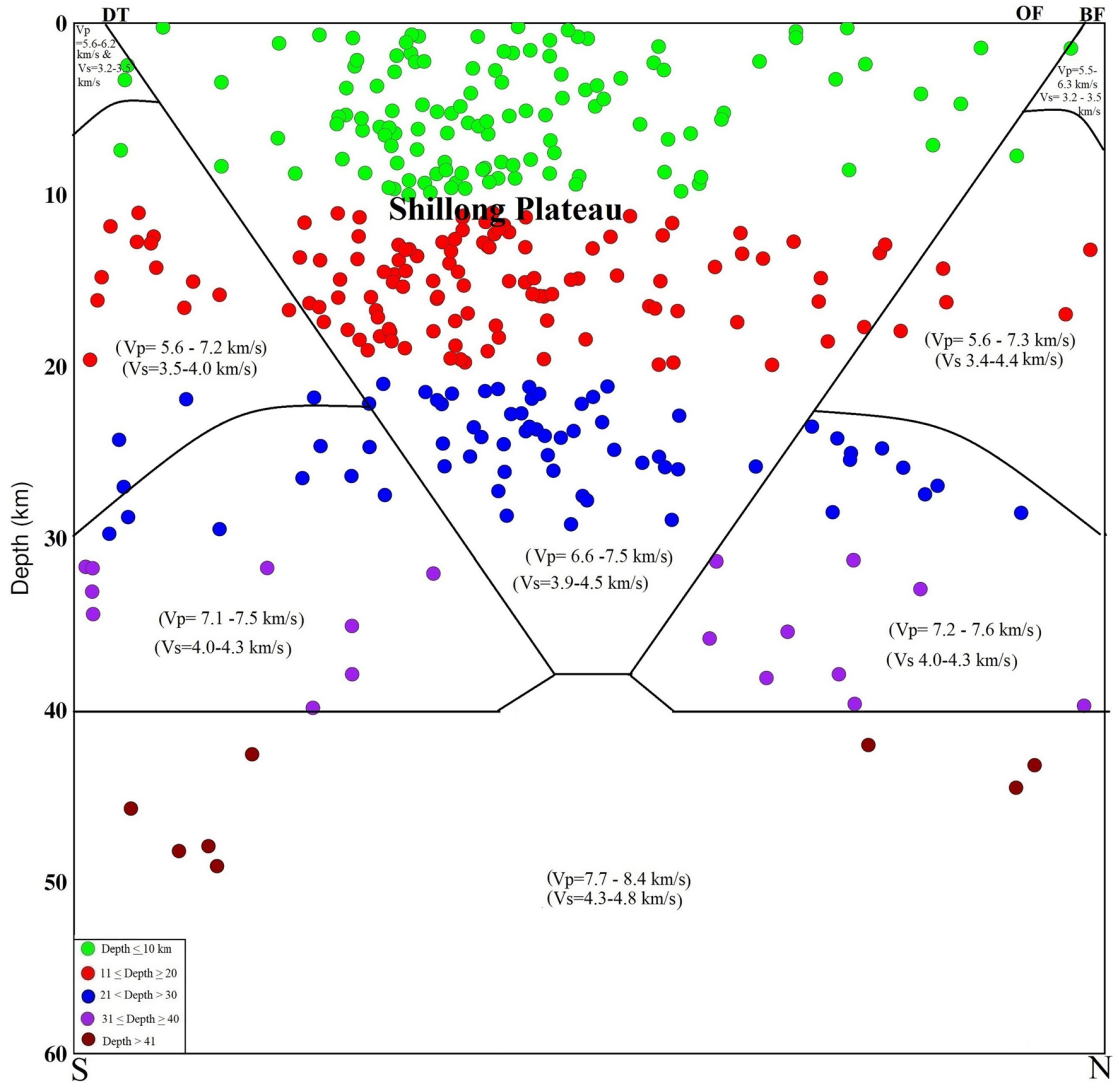
Schematic cross section of a pop-up tectonics beneath the Shillong Plateau, Northeast India region. Circles are the relocated earthquake locations from the present study. The seismic velocities (V_p_ and V_s_) for different layers are also mentioned. The seismogenic faults, namely, the north-dipping DT in association with DF in the south and south-dipping BF in the north located either side to the Shillong Plateau that acted as the causative factors for the pop-up. Uneven distribution of seismic velocities in the upper crust is responsible for earthquake genesis of varying strengths.

Significant lateral heterogeneities in the velocity structures of the crust and upper mantle beneath the plateau are noticed. The lower velocities in the shallower layers are associated with the near surface geology. The velocity reduction near the surface may result from a combination of high sediment thickness, high pore pressure and active strain^27–29^. Our detailed 3-D seismic tomographic assimilation using high-quality phase (P- and S-) arrival time data recorded by the local seismographic network demonstrated that heterogeneities in the crustal faults have contributed significantly to the pop-up tectonics beneath the Shillong Plateau, characterized by high-V and low-σ. In this study, the major geological faults are marked on the top in cross-sections (Figs. 2, 3, 4, 5), and dipping faults at depths are drawn, constraining the seismic velocity anomalies and geological faults. Though, the nature and extent of the crustal heterogeneity and geometry of the seismogenic fault at the source zone, in particular, could not be determined precisely due to several constraints related to grid parameterization and the quality and quantity of the seismic phase data. The north-dipping Dapsi thrust in association with Dauki fault in the south and south dipping Brahmaputra fault in the north located either side to the Shillong Plateau that acted as the causative factors for the pop-up, which attributed to the lithostatic (high-V, low-σ) and sedimentary (low-V, high-σ) load, respectively. The present interpretation of the seismic tomograms taken for different cross-sections (Figs. 2, 3, 4) is further supported by other geological and seismological studies made by other researchers^8,30^. The demarcated faults namely Dapsi thrust in association with Dauki fault and Brahmaputra fault are very much associated with intense seismicity as evident from Fig. 1, but in our cross-sections (Figs. 2, 3, 4), we plotted selected earthquakes in a seismicity width confined to 10 km either side of the fault so lesser number of seismicity at all depth layers in and around the plotted corresponding fault lines are visible in the cross-section. It is so because we wanted to show the distinct boundary of the structural heterogeneities, which is the dictating factor to draw the fault line between the contrast values of velocity and Poisson’s ratio (σ) for better visibility of the contrast having corroboration with earlier geological and geophysical based evidence of fault dispositions^13,14,30^. None-the-less, these observations support the pop-up tectonics of the Plateau advocated by Bilham and England^4^ (2001), Kayal et al.^14^, Rao and Kumar^12^ but not popping up between the Dauki fault and Oldham fault, rather popping up conspicuously between the north-dipping Dapsi thrust in association with Dauki fault in the south and south dipping Brahmaputra fault in the north located either side to the plateau (Figs. 1, 2, 3, 4).

In the uppermost mantle, low seismic velocities and high-σ are quite prominent beneath the plateau. The uppermost mantle is not as seismically active in comparison to the lower crust. The occurrence of earthquakes in the upper crust inferred that high velocity zones are stress accumulators in the strong heterogeneity medium. However, seismic activities along Dapsi thrust and Brahmaputra fault are noticed significantly less during the study period, and it may be inferred that the movement along these faults might be generating compressional stress in the region, thus gyrating the local faults and lineaments to be active and generating seismic activity of smaller magnitudes at shallower depths. The high velocity bodies beneath the plateau, where mostly seismicity is located, indicate that the seismogenic zone that shows brittle behaviour is thicker in this area than other similar tectonics of the regions. A robustness test for this anomaly shows a good recovery of the input structure. This plateau is situated over the buckled-up part of the Indian lithosphere^31^. At depths of approximately 35–50 km, there are narrow low velocities and high-σ below the plateau (Figs. 2, 3, 4). This could indicate that the top of the buckled-up part cracked and was partially underthrusted by low-velocity materials. The low velocity towards the northern part of the region may not put up much resistance to underthrusting, leading to less seismic activity. Within the plateau region, N-S trending low V_p_, V_s_ and high-ϭ zones could be associated with the faults and lineaments present in this area^4^. It is also noticed that an approximately 200 km long E-W Dauki fault is well imaged, indicating high velocities and low-ϭ towards the western region and low- velocities and high-ϭ on the eastern sides (Figs. 2, 3, 4).This evidence of a subducting Indian plate is noticeably imaged as high velocities and low σ. It is striking that these seismic velocity structure variations in the crust are localized and isolated. The great earthquakes that occur in the upper crust suggest that a strong anisotropy is developed in response to the shear processes responsible for the occurrence of great earthquakes in that sector of the plateau. Beyond 60 km, we have no better resolution of the assimilated seismic structures because of the reduced spatial distribution of criss-crosses among seismic rays at deeper depths (Supplementary Fig. S4a,b). Despite the high tectonic complexity of the region, some correlation may be highlighted between geological units and velocity structures. In some of the profiles, the high velocities and low ϭ ratio penetrate the upper crust and dip northwards, where the thickness of the crust varies vigorously. Consequently, the low-σ perturbations suggest that the low value could have been due to fewer pores and competent rock, which can store stress accumulation to a greater extent. On the other hand, the high-σ in the lower crust or in some patches may suggest relatively weaker rocks of the subsurface, which can undergo metamorphism processes at deeper layers under suitable pressure and temperature or the presence of aqueous pore fluids^32,33^. This high-σ may have facilitated the process of brittle failure at depth by relaxing the seismogenic zones with the release of accumulated stress in metamorphic rocks. The seismic velocity profiles along the A–B, C–D and E–F at depths of 5–10 km is conspicuously characterized by a low velocity zone and high-ϭ, which is due to the presence of sedimentary sequences deposited in the marine environment. Our σ estimates at these depth ranges also provide a good constraint on our interpretations. Furthermore, the Oldham fault is more or less flat at depth toward the aseismic semi-brittle zone below the seismogenic layer (Figs. 2, 3, 4). Flatten in the mid crust region and detach at the top of the aseismogenic layer. Beneath the plateau, the thickness of the crust is over ~ 45 km around the Moho, and a high velocity zone obviously exists^10,34^. In comparison Brahmaputra fault and Dapsi fault have a strong impact on the velocity structures in the middle and lower crust. As the crust thickens beneath the plateau, pressure increases can trigger metamorphic reactions at lower crustal levels; in particular, evidence for transformation of eclogite in the Indian plate provides support for theories about mass transfer from the lithosphere to the underlying mantle^26,35^. The low-σ, high conductivity and earthquakes are consistent with the presence of fluids with high pore pressure in highly fractured materials. As Lemonnier et al.^36^ suggest, fluids can originate from dehydration reactions in the Indian crust under thrusts beneath the Himalaya. In this study Moho configuration is clearly resolvable.

The plateau is characterized by positive Bouguer and isotstaic anomalies, which imply denser and thicker crust beneath the plateau^37^. Several faults are encountered in the complexity zones. As a result, crustal structures are complicated, and strong lateral heterogeneity exists in the crust and upper mantle. It is also interesting to note that very few earthquakes are located in the depth range of 30–60 km, which mainly occurred in the high-ϭ zone. This again suggests that earthquakes in this depth range are associated with ductile parts of underneath rock materials. We may interpret that the upper mantle materials act as jelly, as proposed in the jelly sandwich model of the seismogenic zone, which is anticipated by Chen and Molnar^7^ for continental crustal seismicity. Poisson’s ratio (σ) is more diagnostic of crustal and subcrustal rock properties and has a very close bearing on the seismogenic strengths and material properties of in-situ rock beneath the study area and its adjoining areas. It is important to mention a high σ suggests weak and less competent rock materials associated with fractures/cracks. The fractures and cracked volume of rocks in the tectonically complex zone may be partially or fully saturated at the subsurface layers, which require detailed geotectonic information about the area for compressive interpretation.

The plateau is associated with several seismotectonic faults, which may have caused fracturization of underlying rocks. This region where the great 1897 Shillong earthquake occurred indicated that the earthquake in the region contributed to the relaxation of high ambient stresses that were locally concentrated within rheological heterogeneities and the relatively competent and crystalline parts of rock materials beneath the study area. This region contains highly fractured and faulted granitic bodies at the surface^14,38^. This plateau may accommodate significant strain due to the compression of faults from all sides.

A closer examination of the distribution of the P- and T -axes suggested that although the pattern seems quite variable, there is a consistent picture of an overall 5 mm/year shortening taken up across the plateau and its borders^39^. Supplementary Fig. S5 shows a prevailing strike-slip environment in the Shillong region with both P- and T-axes oriented sub-horizontally. The distributions of P- and T-axes are also inferred a more variable pattern along the Oldham fault, Barapani Thrust and Kopili fault, perhaps owing to the strong heterogeneities along faults (Supplementary Fig. S5). The schematic cross section of a pop-up tectonics beneath of the Shillong Plateau, Northeast India region of the plateau is summarized in Fig. 5. The pop-up tectonics is confirmed by present seismic tomography study. This plateau shows high-velocity bodies < 40 km depth where 76% seismicity is observed, indicating a seismogenic zone. The brittle behaviour is thicker in this area than what is observed by many researchers in other parts of NE India.

The generation of a shallow crustal earthquake could be controlled by a deep process in the lower crust and upper mantle. It is vital to investigate the detailed structure and processes of the lower crust and upper mantle to clarify the seismogenesis and reduce earthquake risk. It is insufficient to refer only to the surface description of spatial features to predict the seismic potential of a region. Large damaging earthquakes occurred in décollement zones that leave very little surface evidence of faulting that can be used to identify past earthquakes. We infer that the use of detailed 3-D seismic tomography may offer potential information on Pop-up tectonics beneath the Shillong Plateau to unravel what and how the genesis of such large damaging earthquakes caused. This study may help in evolving a comprehensive earthquake hazard mitigation model for a region.

## Methods

### Date and algorithm for Earthquake Tomography

To understand the seismicity pattern in the active part of the Shillong Plateau, this is still poorly instrumented to obtain detailed seismological investigation. A Geological Survey of India (GSI) conducted earthquake monitoring from March 2010 to February 2013 by setting up a total of 11 temporary 3-component digital broadband seismographs in a network (Fig. 1). Among which, a total of three short-period seismographs with 3-components were also installed for precise detection of direct P- and S-arrival times of earthquakes that occurred within 25–30 km. The seismographs were operated by a rechargeable 12-V power-safe battery of 100 amperes per hour associated with an inbuilt GPS to obtain precise locations of seismographs with the reference time of the event. The phases that were compiled in the monthly seismological bulletin of the NCS data recorded at 5 permanent broadband seismic stations from March 2010 to December 2014 were also used in this study. The present analysis is based on the acquisition of seismological data through the campaign mode and permanent stations. All seismic stations were equipped with a three-component seismograph. These stations recorded 40–100 samples per second. As shown in Fig. 1, this seismic network consists of 16 seismographs covering the whole Shillong Plateau. The picking accuracy of arrival times is estimated to be ~ 0.03–0.12 s for the P-waves and ~ 0.06–0.22 s for the S-waves. Between March 2010 and December 2014, a total of 1000 events with magnitude M ≥ 1.8 were recorded by temporary and permanent seismic networks of the Shillong region. We employed the HYPO71 algorithm^40^ to determine the locations of the events. The located events with root mean square error (rms) ≤ 0.2, horizontal error ≤ 1.5 km, vertical error ≤ 2.5 km a minimum of 4-S arrival times to better constrain the depth. These databases have been scanned with two main criteria: an event recorded by at least four seismic stations with four reliable P- phases and at least two reliable S-phases are considered. Second, the initial locations of the events are located with rms ≤ 0.1 s. This procedure reduced the dataset to 670 events, including 3798 P- and 3567 S-waves, which were used for simultaneous inversion (Supplementary Fig. S6). The ray tracing of events at different depths and depths are depicted in Fig. S4b. The plot between the travel time (s) and epicentral distance (km) (a) P-wave and (b) S- wave travel time used in the present study is shown in Fig. S6. We tested different available velocity models^10,17,18,27,34,38,41^. Finally, we selected Mishra et al.^10,27^ models based on residuals.

We employed the 3-D tomographic technique of Zhao et al.^42^ to determine the 3-D velocity structures of the Shillong Plateau in the present study. It is an iterative algorithm for simultaneous inversion of P- and S-arrival times from local and regional events resulting in three-dimensional distributions of seismic velocities (V_P_ and V_S_) and source coordinates. After obtaining the V_P_- and V_S_- seismic wave velocity model, the relation (Vp/Vs)^2^  = 2(1–σ)/(1–2σ) is used to determine the elastic parameter, Poisson’s ratio^43^ (σ). The cross-sectional Poisson’s ratio (σ) images are shown in Fig. 4. According to the ray density distribution, we parameterized this study volume using a grid of node distributions, following the Zhao et al.^42^ algorithm. To calculate ray paths and arrival times precisely and rapidly, the efficient 3-D ray tracing algorithm of Zhao et al.^42^ is applied that iteratively uses the pseudo bending method of Um and Thurber^44^ and Snell’s law to search the fastest ray from an initial estimate of the ray path. We used some events outside of the network, which could significantly improve the results of local earthquake tomography^45^. To avoid large model variations, a suitable damping parameter is selected for the diagonal elements of the separated medium matrix that results in near-zero singular values. We selected the damping parameter by conducting a series of iterative inversions using a large range of damping values based on studies performed by other researchers using the 3-D tomographic method^46^. The norm of solutions (%) versus rms travel time residual to select a damping parameter of 10 for P- and S-wave velocity in this study (Supplementary Fig. S7). The selected value of damping has shown reasonable control on estimated velocity anomalies that minimize the influence of seismic noise in the data.

### Resolutions tests

We rigorously conducted board resolution tests for both V_p_- and V_s_- seismic structures using the checkerboard resolution test method of Zhao et al.^42^, originally derived from Humphreys and Clayton^47^ and Inoue et al.^48^, to examine the spatial resolution of the present dataset. We have done a series of checkerboard tests. The results of these tests have shown that anomalies smaller than 20 km can only be resolved in the study area where most seismicity and seismic stations are located. In most parts of the study area, such anomalies are strongly replotted due to the dominance of orientations of seismic rays due to the enhancement of criss-crosses along seismic rays between sources to receivers. However, for anomalies of 25 km size, the situation improves. Finally, anomalies of 25 km size are robustly resolved throughout the study area (Fig. S8). This test allows us to distinguish the anomalies by size and location and helps us to estimate which of them are robust. Based on these tests, we defined the resolved area; outside, the resulting anomalies were masked. The resolution test with a grid spacing of 25 km indicates good resolution for both V_p_ and V_s_ anomalies beneath the Shillong Plateau. The checkerboard resolution tests for both V_p_ and V_s_ are shown in Fig. S8a,b. This test is carried out to assess the resolvability of data used in assimilating the velocity structures beneath the study area for the given model parameterization and it also helps to understand how much recovery of current velocity anomalies is possible within the model for given grid spacing set up for both lateral and vertical directions. This test allows us to distinguish the anomalies by size and location and helps us to estimate which of them are robust. Based on these tests, we defined the resolved area; outside, the resulting anomalies were masked. The tomograms are assimilated for different depth slices for V_p_- and V_s_ structures. We tested our dataset for different grid spacings in both the lateral and vertical directions of the model space by keeping the preamble of resolution, as the grid spacing should be smaller than the minimum resolved size of velocity anomalies at every pixel (Fig. S2). We found that our 3-D seismic tomograms (V_p_, V_s_, σ) are well resolved for the grid setup.

We provided both positive and negative velocity perturbations with a 5% anomaly alternatively to the grid nodes in the entire 3-D grid setup. We achieved the results of the checkerboard resolution test at the different representative layers for V_p_ and V_s_ as shown in Fig. S8a,b. The checkerboard resolution test is found to recover 3% of anomalies for most of the layers except for the edge part of the study area, which has less crisscross among seismic rays for the given model parameterization. We conducted a series of synthetic tests with different sizes of anomalies. Based on the recovery results, we defined a zone with satisfactory resolution. In this presentation, the main results of the tomography inversion are shown by masking edge portions.

Furthermore, to understand resolution through the recovery of the velocity anomalies for the Shillong plateau, we conducted model recovery synthetic tests. We designed the background model shown in Fig. S9a is similar to the inverted image of the Shillong Plateau shown in Fig. 2a at a depth of 30 km. Figure S9b,c show the inverted results, indicating the pattern of velocity anomalies is well recovered at all depths. In the western part of the model, the change of signs of anomalies is correctly resolved, and the positive and negative anomalies are recovered at correct locations. However, the recovery of the velocity amplitudes is poor under the edge portion due to the lack of ray paths there. We found the velocities heterogeneities are well recovered through the amplitude of the velocity perturbation are smaller than that in the input model. The high and low velocity zones are well resolved at all depths.

While conducting tests with both checkerboard and recovery or restoration, we carefully tuned the controlling parameters to reproduce the conditions of the observed data experiment, as closely as possible, by using the same ray geometry, perturbing the data with realistic noise and performing the calculations according to the same workflow. Thus, the control parameters optimized for synthetic models appear to be suitable for the case of experimental data processing. Therefore, the final tomographic model was calculated using the parameters determined in checkerboard tests and synthetic modelling to achieve the best recovery of models.

## Supplementary Information

Supplementary Figures.

## Data Availability

We used seismic waveform data recorded during the period 2007–2009 by the seismographic network ascribed to the Geological Survey of India. We also used seismic phases that compiled in the monthly seismological bulletin of the NCS data recorded at 5 permanent broadband seismic stations from March 2010 to December 2014 were also used in this study. Some figures were prepared using public domain GMT software^49^.
