# Supplementary material for: Seismic evidence of pop-up tectonics beneath the Shillong Plateau area of Northeast India
Source: Sci Rep. 2022 Aug 19;12:14135. doi: 10.1038/s41598-022-18389-0 (PMC9391424; doi:10.1038/s41598-022-18389-0)
Supplement: Supplementary file 1 — Supplementary Figures. [file 41598_2022_18389_MOESM1_ESM.docx]

**Seismic evidence of Pop-up tectonics beneath the Shillong Plateau area of Northeast India**

A. P. Singh^1^, O. P. Mishra^1*^, O. P. Singh^2^

**Supplementary Information** includes 9 additional figures supporting the main results of the paper

Figure S1. (a) Map shows the epicentral area impacted during the 1897 Shillong earthquake marked by the blue curvilinear lines centered on the Shillong Plateau reported by Oldham’s (1899), and a red rectangle indicating the inferred 1897 subsurface rupture. The star indicates the vertical projection of the source of the 1897 Shillong earthquake on the surface having 26^o^N; 91^o^E of the Oldham (1899). The focal mechanism of the 1897 Shillong earthquake is also shown. The abbreviations of faults correspond to fig. 1.

Figure S2: Vertical cross sections of the Vp/Vs. The positions of cross sections are shown in Figure 1. The star shows 1897 Shillong earthquake (M_s_8.7). The geological faults are also marked.

Figure S3a: Maps showing the perturbation of P-wave velocity in % at different depth levels such as 10 km, 20 km, 30 km, 40 km, 50 km, and 55 km. The layer depth is indicated on the left side of each map, and the thin lines depict the faults and lineaments. The circles indicate the earthquakes.

Figure S3b: Same as Fig. 5a but for S-wave velocity.

Figure S4a: The spatial distribution of seismic ray paths at different depths that are used in the tomographic study. Every raypath between an epicenter and a station is drawn as one straight line. Red triangles show seismic stations that recorded the arrival times.

Figure S4b: Maps showing the distribution of depthwise raypaths connecting different sources corresponding to different receivers also associated with many of the dense raypaths in the plot exhibits the darkest zones at various depths. The solid circles depict the distribution of events. The positions of cross-sections are given in Fig.1.

Figure S5: Horizontal projections of the P- and T axes of the 41 available focal mechanism solutions. Red and blue line segments are for the P- and T-axes, respectively, and the yellow dots are epicentral locations. The length of each projection is determined by the plunge of the axis, with the maximum length corresponding to a plunge angle of 0°, i.e., a horizontal axis. Black lines are faults as introduced in Fig. 1. White circles show seismicity in the Shillong Plateau.

Figure S6: Plot between the travel time (s) and epicentral distance (km) (a) P-wave and (b) S- wave travel time used in the present study. The plots have about 3798 P and 3567 S arrival times from 669 events.

Figure S7: Trade-off curve between the norm of solution and the root-mean-square (RMS) travel-time residuals (a) for P -wave velocity and (b) S-wave velocity. Numbers along the curves represent the damping parameters. The optimal damping parameter (10.0) is determined by obtaining our preferred tomographic model.

Figure S8a: The results of the checkerboard resolution test for P-wave velocity at six depths. Blue and red patches denote low and high velocities, respectively. The perturbation scale is shown at the bottom. Maps also showed the pattern of recovery in black and red patches of negative and positive perturbations of different extent of recovery, respectively. The size of the patches represents the extent of the recovery. The variation in the size of the patches represents variation of recovery between +3% and -3%. It is noticed that the edge of the tomograms at each depth and down to depth > 40 km are representing smearing.

Figure S8b: Same as figure S5a but for the results of the checkerboard resolution test for S-wave velocity.

Figure S9: Results of the synthetic recovery-test (a) input model and (b) Vp- (c) Vs- inversion results determined by using local data. Red color depicts low velocity while blue color depicts high velocity. The velocity perturbation scale is shown at the bottom.


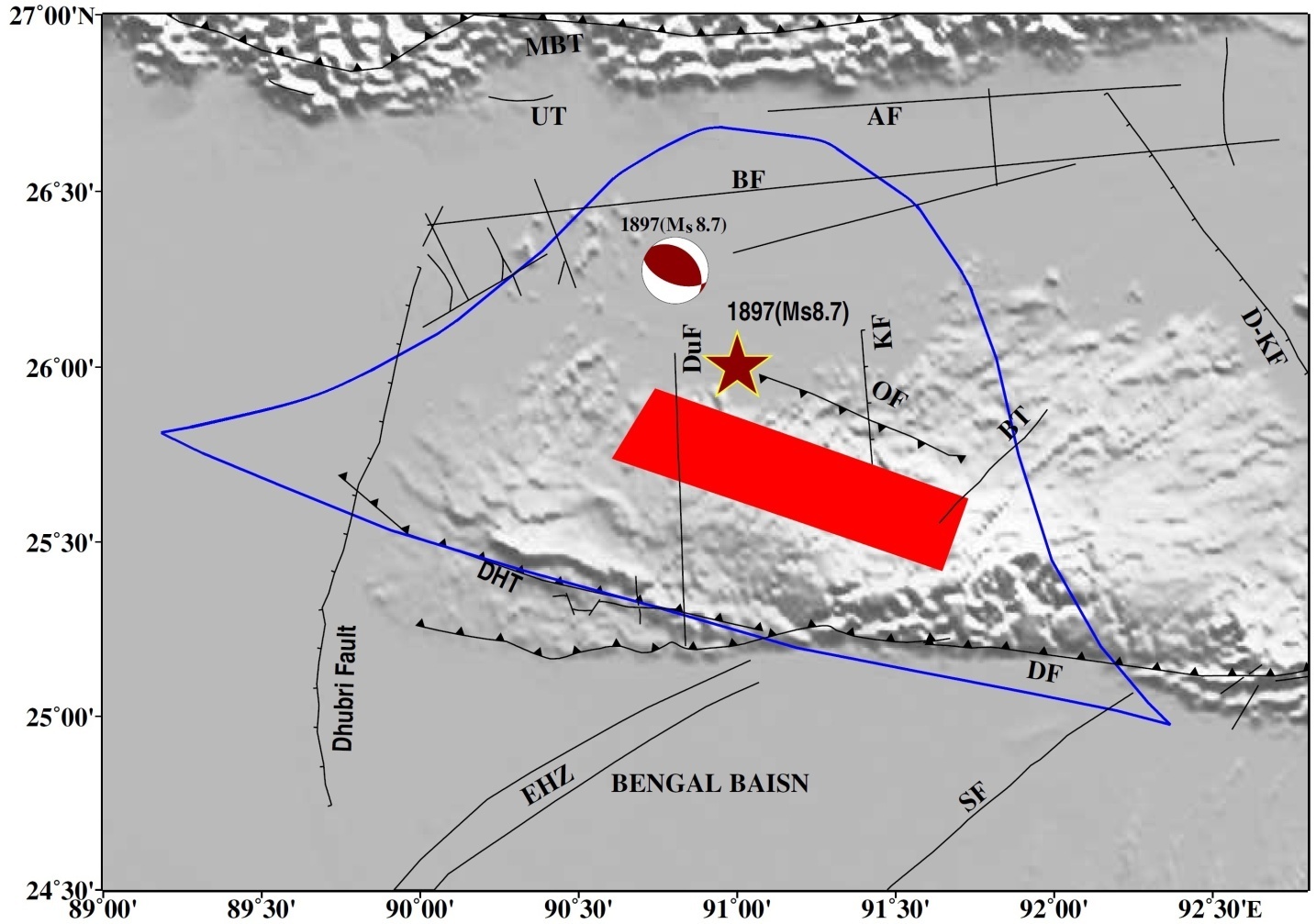


Figure S1. (a) Map shows the epicentral area impacted during the 1897 Shillong earthquake marked by the blue curvilinear lines centered on the Shillong Plateau reported by Oldham’s (1899), and a red rectangle indicating the inferred 1897 subsurface rupture. The star indicates the vertical projection of the source of the 1897 Shillong earthquake on the surface having 26^o^N; 91^o^E of the Oldham (1899). The focal mechanism of the 1897 Shillong earthquake is also shown. The abbreviations of faults correspond to fig. 1.


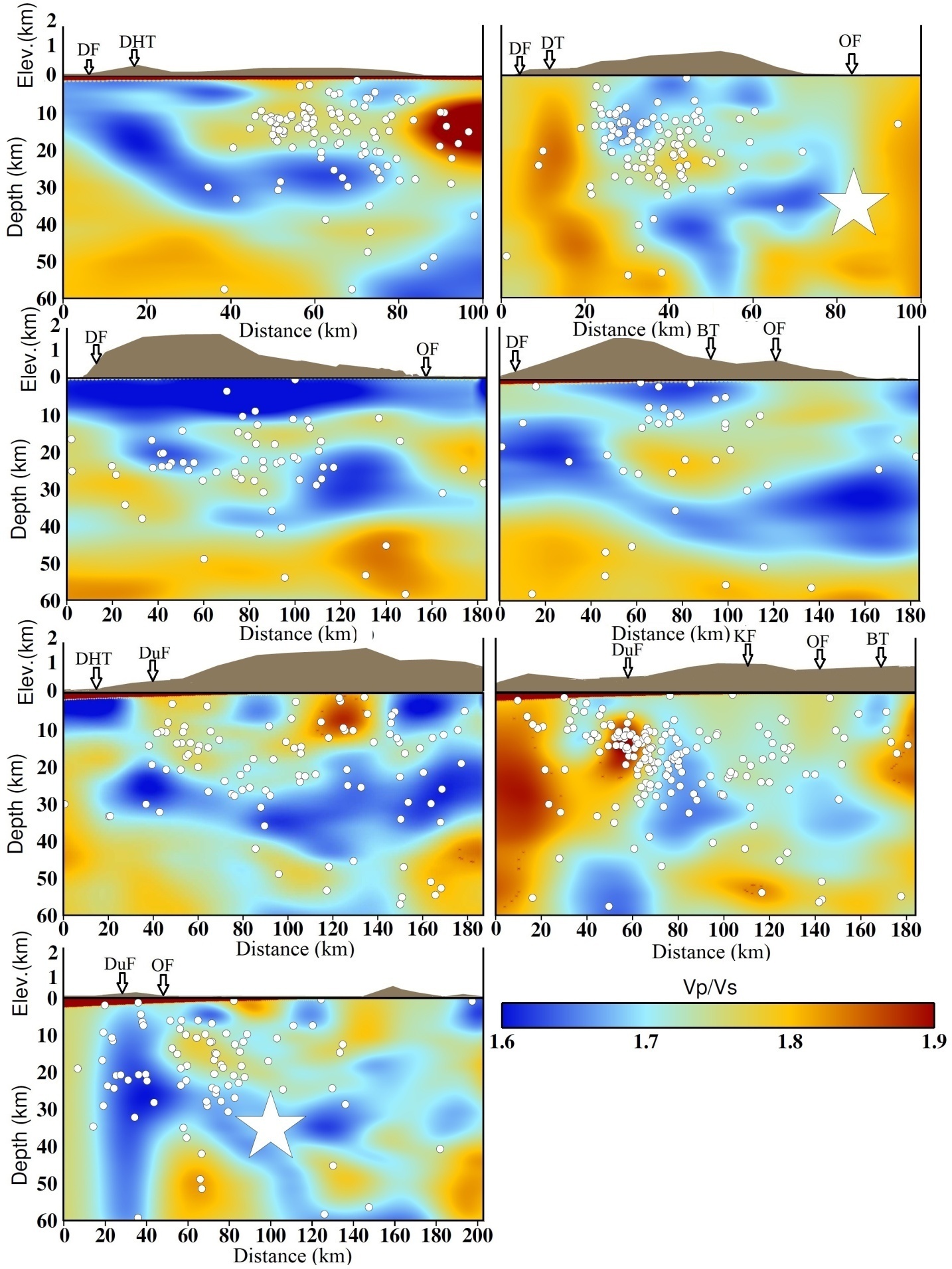


Figure S2: Vertical cross sections of the Vp/Vs ratio. The positions of cross sections are shown in Figure 1. The star shows 1897 Shillong earthquake (M_s_8.7). The geological faults are also marked


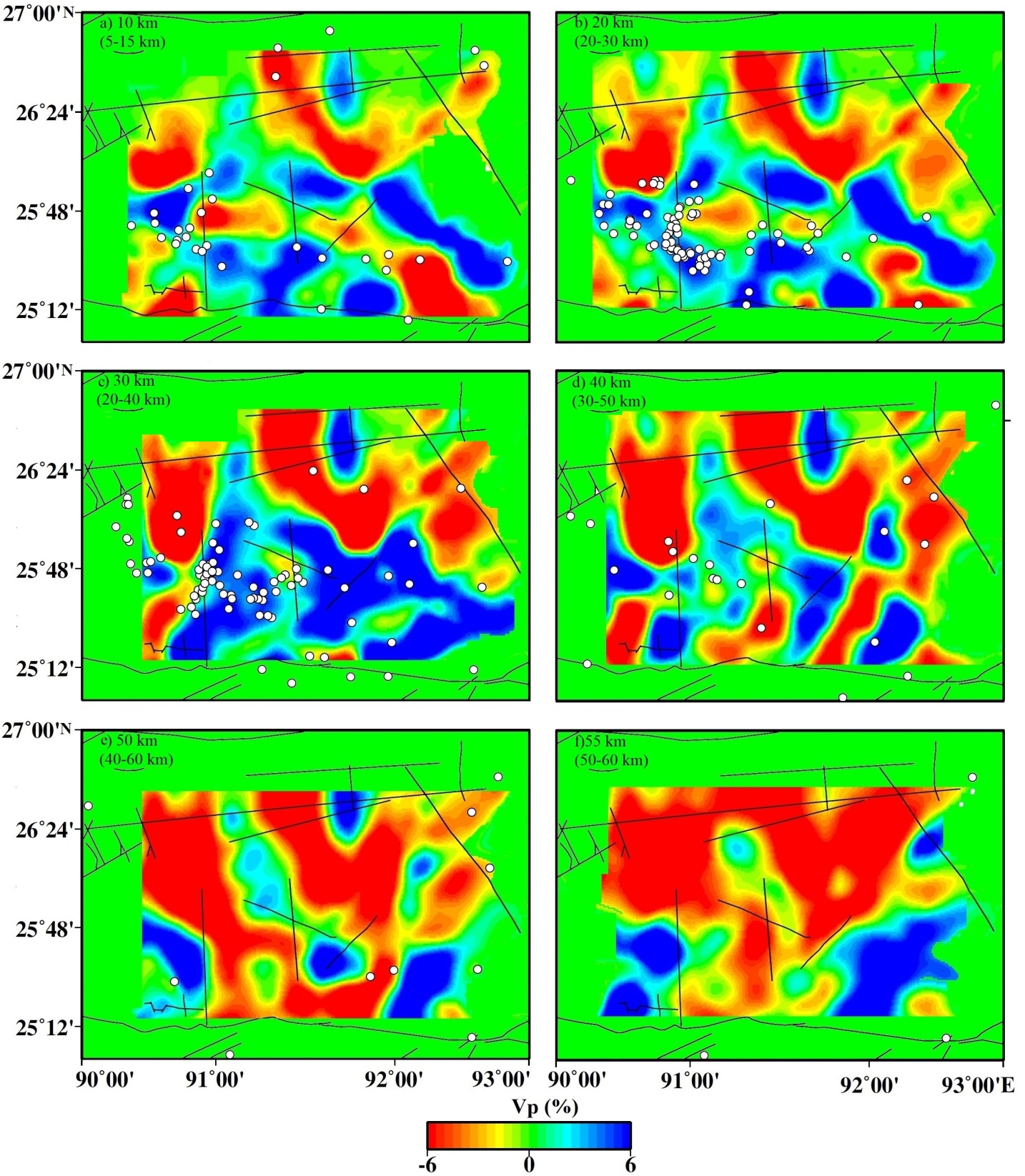


Figure S3a: Maps showing the perturbation of P-wave velocity in % at different depth levels such as 10 km, 20 km, 30 km, 40 km, 50 km, and 55 km. The layer depth is indicated on the left side of each map, and the thin lines depict the faults and lineaments. The circles indicate the earthquakes.


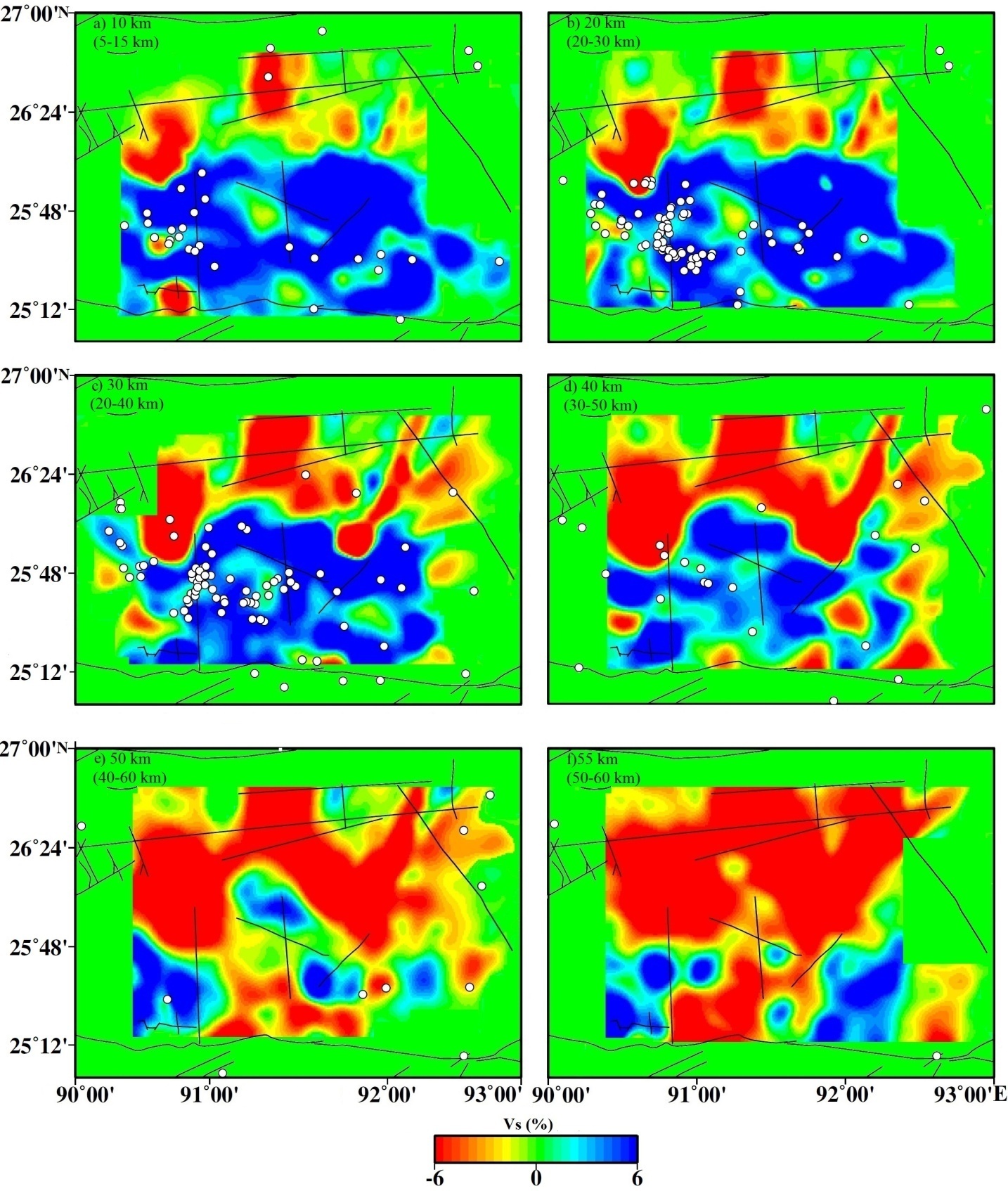


Figure S3b: Same as Fig. 5a but for S-wave velocity.


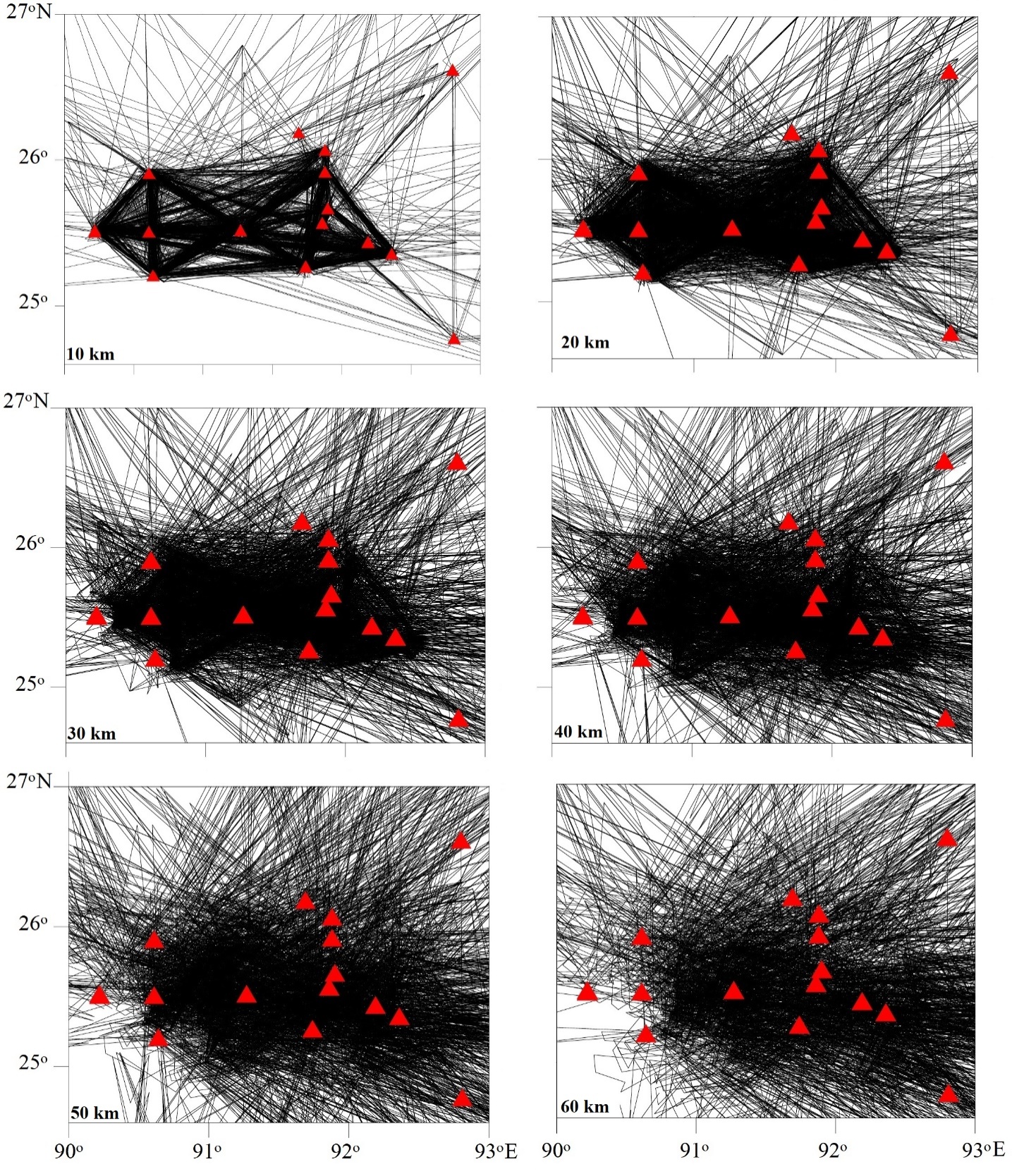


Figure S4a: The spatial distribution of seismic ray paths at different depths that are used in the tomographic study. Every raypath between an epicenter and a station is drawn as one straight line. Red triangles show seismic stations that recorded the arrival times.
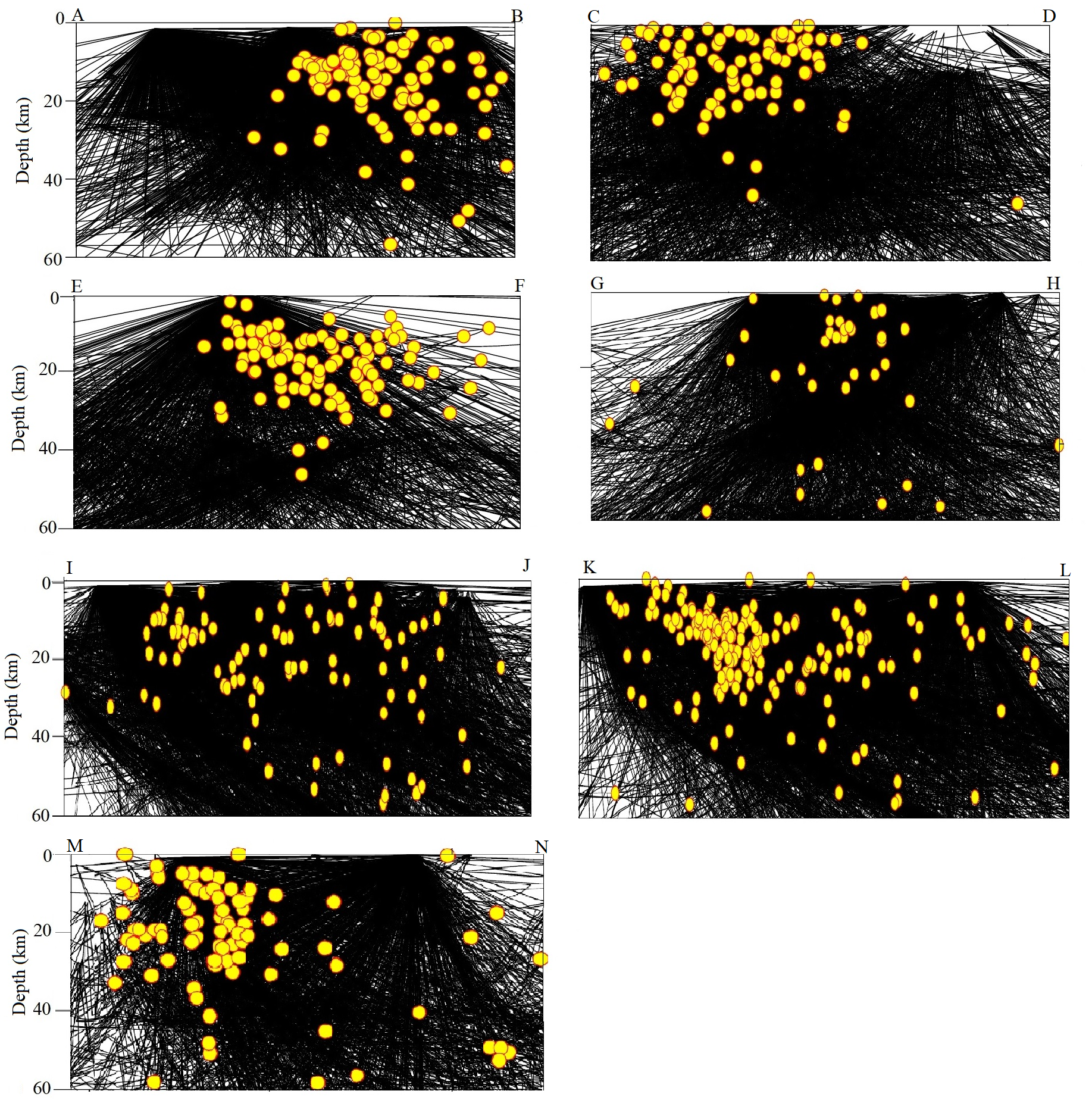
Figure S4b: Maps showing the distribution of depth wise raypaths connecting different sources corresponding to different receivers also associated with many of the dense raypaths in the plot exhibits the darkest zones at various depths. The solid circles depict the distribution of events. The positions of cross-sections are given in Fig.1.


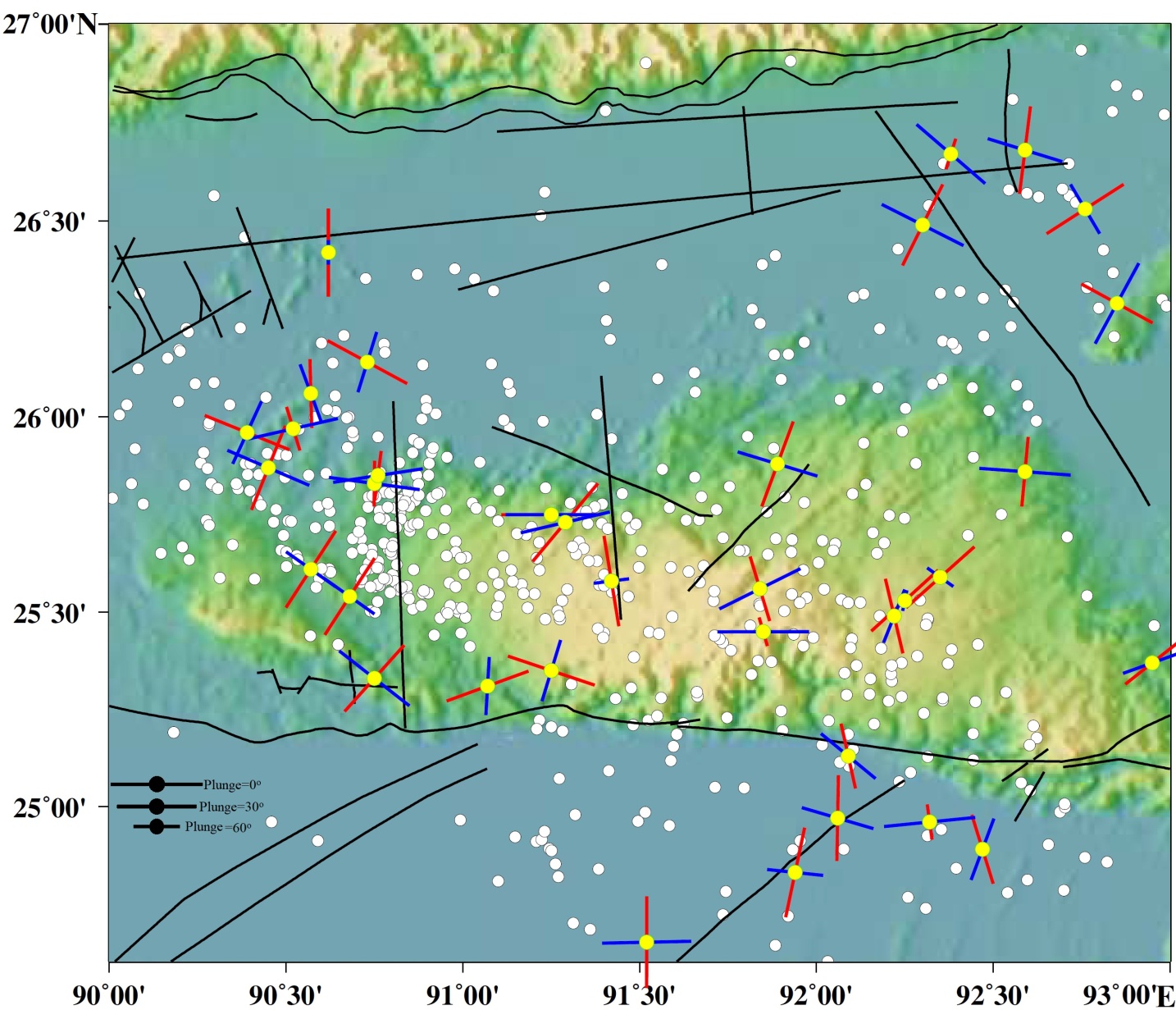


Figure S5: Horizontal projections of the P- and T axes of the 41 available focal mechanism solutions. Red and blue line segments are for the P- and T-axes, respectively, and the yellow dots are epicentral locations. The length of each projection is determined by the plunge of the axis, with the maximum length corresponding to a plunge angle of 0°, i.e., a horizontal axis. Black lines are faults as introduced in Fig. 1. White circles show seismicity in the Shillong Plateau.


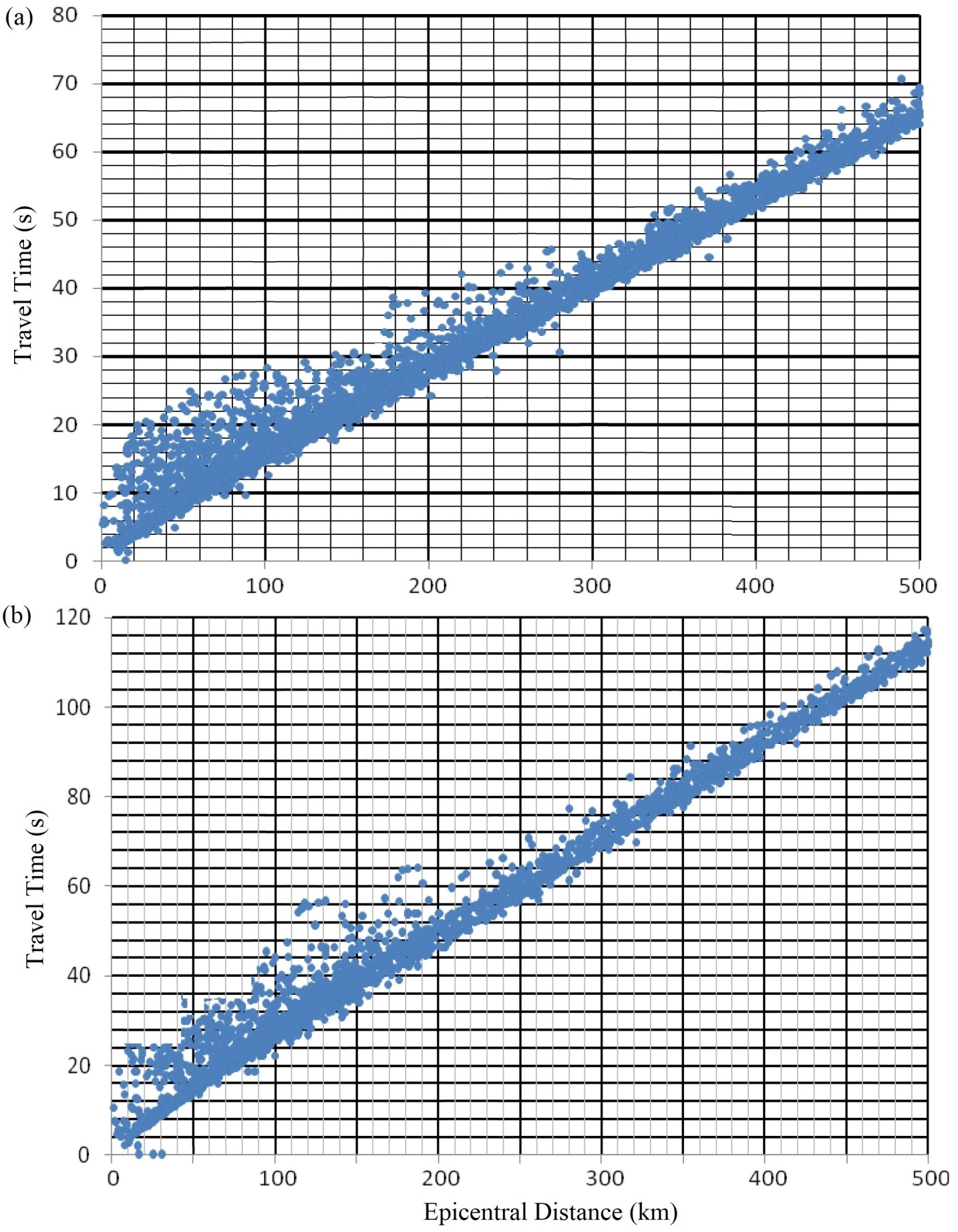


Figure S6: Plot between the travel time (s) and epicentral distance (km) (a) P-wave and (b) S- wave travel time used in the present study. The plots have about 3798 P and 3567 S arrival times from 669 events.


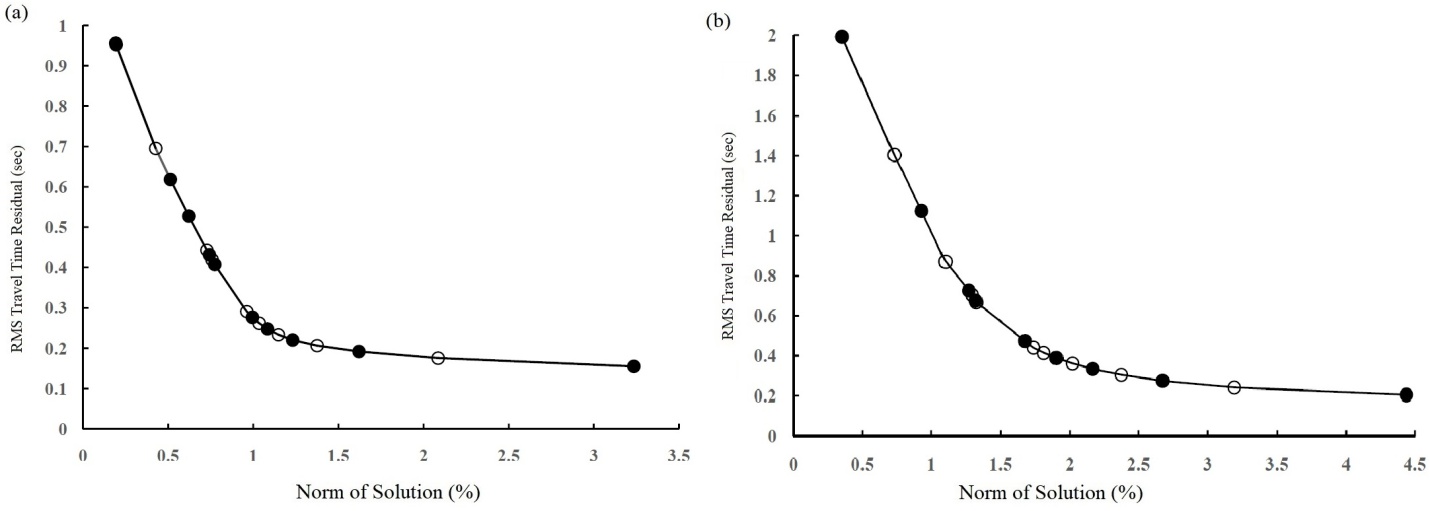


Figure S7: Trade-off curve between the norm of solution and the root-mean-square (RMS) travel-time residuals (a) for P -wave velocity and (b) S-wave velocity. Numbers along the curves represent the damping parameters. The optimal damping parameter (10.0) is determined by obtaining our preferred tomographic model.


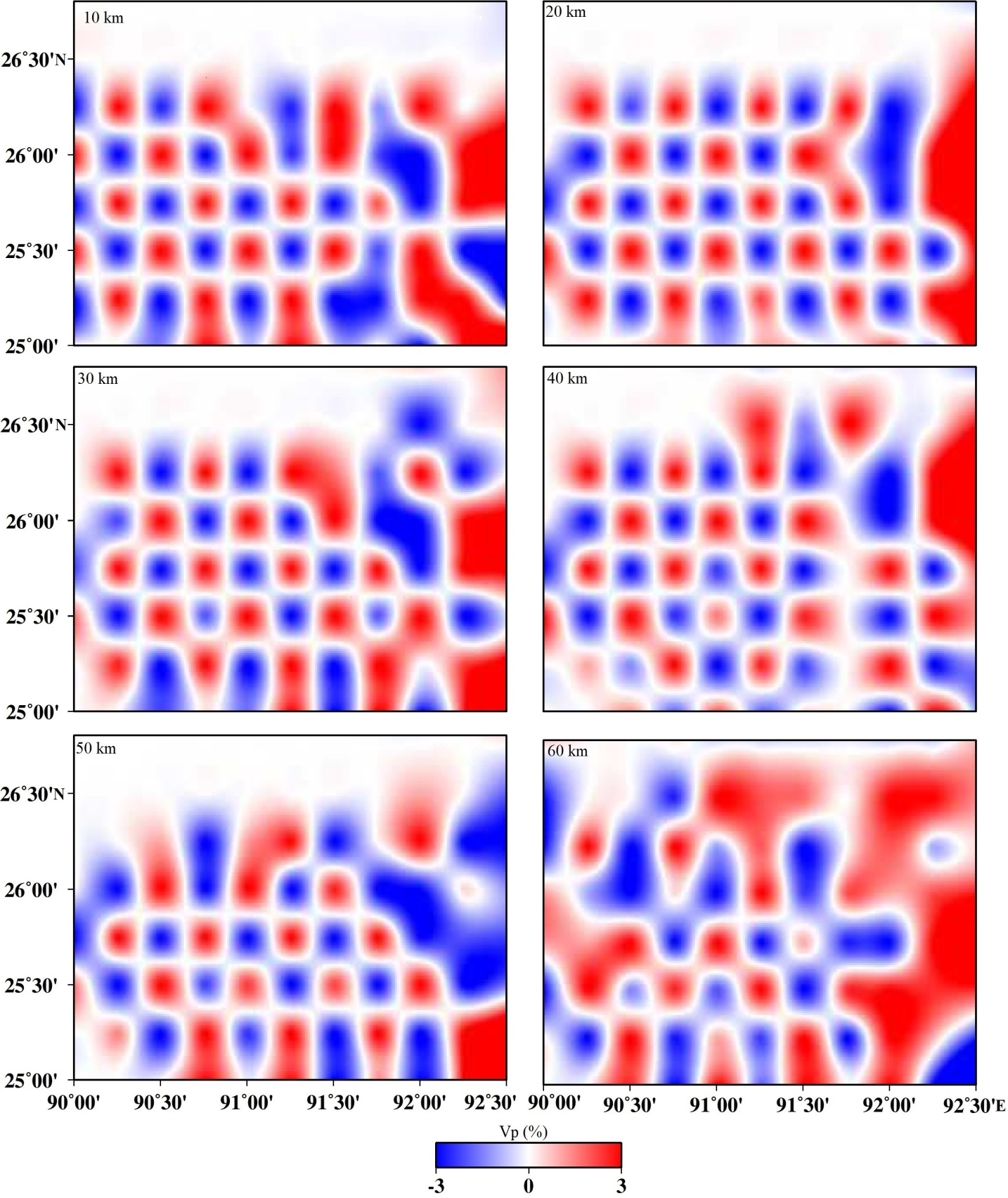


Figure S8a: The results of the checkerboard resolution test for P-wave velocity at six depths. Blue and red patches denote low and high velocities, respectively. The perturbation scale is shown at the bottom. Maps also showed the pattern of recovery in black and red patches of negative and positive perturbations of different extent of recovery, respectively. The size of the patches represents the extent of the recovery. The variation in the size of the patches represents variation of recovery between +3% and -3%. It is noticed that the edge of the tomograms at each depth and down to depth > 40 km are representing smearing.


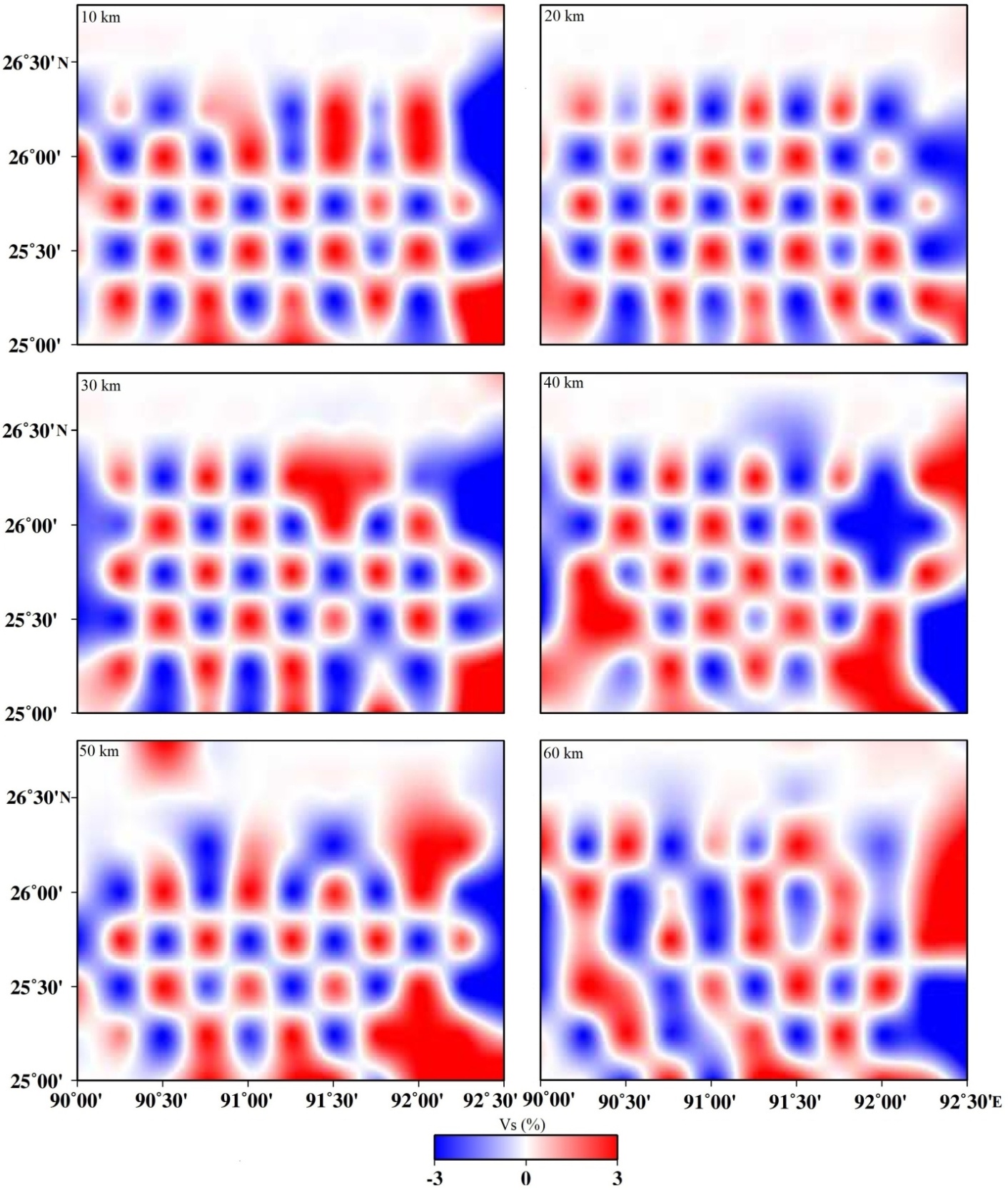


Figure S8b: Same as figure S5a but for the results of the checkerboard resolution test for S-wave velocity.


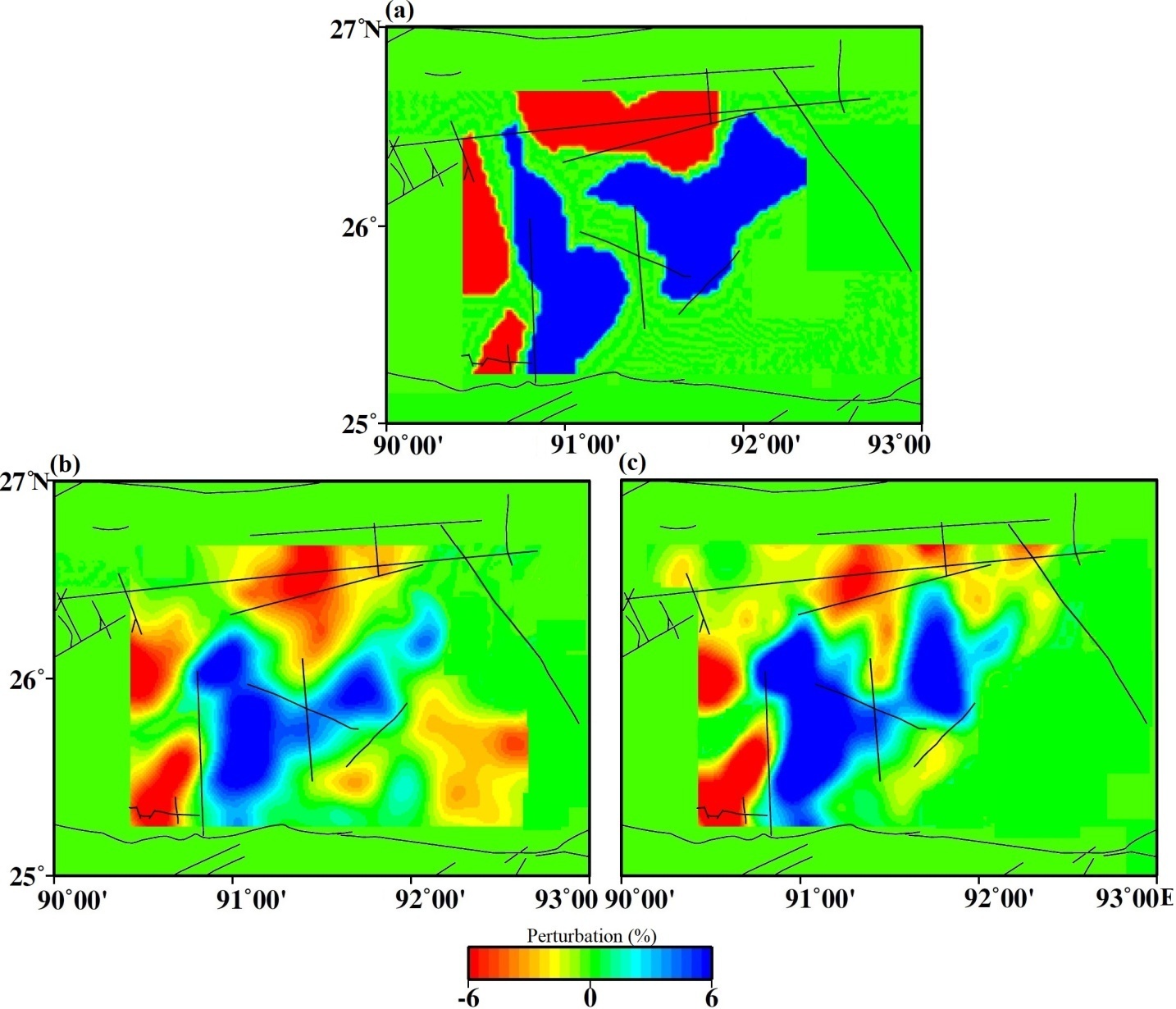


Figure S9: Results of the synthetic recovery-test (a) input model and (b) Vp- (c) Vs- inversion results determined by using local data. Red color depicts low velocity while blue color depicts high velocity. The velocity perturbation scale is shown at the bottom.
